# Analysis of the performance of a new concept for automatic draping of wide reinforcement fabrics with pre-shear: A virtual prototyping study

**DOI:** 10.1016/j.heliyon.2023.e20263

**Published:** 2023-09-22

**Authors:** Christian Krogh, Peter H. Broberg, Sebastian M. Hermansen, Asbjørn M. Olesen, Brian L.V. Bak, Esben Lindgaard, Erik Lund, Jørgen Kepler, Johnny Jakobsen

**Affiliations:** a Department of Materials and Production, Aalborg University, Fibigerstraede 16, 9220 Aalborg, Denmark; b CraCS Research Group (cracs.aau.dk), Aalborg University, Fibigerstræde 16, 9220 Aalborg, Denmark

**Keywords:** Draping, Finite element modeling, Wind turbine blades, Laminated composites, Glass fiber fabric

## Abstract

The layup process of large composite structures made from dry reinforcement fabrics is considered. One such structure is a wind turbine blade, for which the current draping process is mostly manual. Automating the draping process will, therefore, lower the costs. Based on a literature review, a new concept is synthesized and analyzed using an advanced finite element model with rigid multi-body kinematics and a dedicated material model for the fabric. The material model is calibrated using experimental coupon tests, i.e. the bias-extension test (shear) and Peirce's cantilever test (out-of-plane bending). The concept is analyzed numerically by means of a simple parameter study and draping test cases on a flat mold as well as a general double-curved mold. The simulation results show that the concept is feasible for the draping operation and is thus qualified for the subsequent physical prototyping.

## 1 Introduction

Laminated fiber-reinforced composite materials offer a superior balance between weight and mechanical performance. They are found in an increasing number of applications within fields such as aerospace, automotive, maritime, sporting goods, and wind turbine blades. Regarding the latter, a rapid growth in size is currently taking place, which challenges the manufacturing process and the associated costs. Trends in wind turbine blade manufacturing focus on the exploration of *preforms*, i.e. smaller pre-fabricated stacks of fiber material formed to near-net shape and implementing automation processes. One thing to note, is that the fabric material must accommodate local in-plane shear to adapt to a general double-curved mold surface as discussed by Cao et al. [1]. The draping operation is, therefore, more than just a pick-and-place operation.

In general, different concepts for automating the layup of fiber plies have been developed. Very general and versatile systems like automated tape laying (ATL) and automated fiber placement (AFP) exist. While they can deposit material at over 70 m/min, they work with narrow prepreg-based tapes, and the tooling and path-planning are complicated as discussed by Lukaszewicz et al. [2]. On the other hand, specialized concepts exist, that have been built for specific combinations of fabric material, mold curvature and mold size. For aerospace applications, see e.g. Elkington et al. [3], Szcesny et al. [4], and Ellekilde et al. [5], with other systems discussed in the review paper by de Zeeuw et al. [6].

Wind turbine blades are long structures (currently 100+ m for a typical modern offshore blade) and made from thick non-crimp fabric (NCF), but the shear angles involved in the draping of the fabric are typically low. Different concepts have been developed to accommodate these conditions, and typically take the form of a gantry with a layup head continuously draping the fabric from a feed roll, see, for instance, Weigel & Müller [7], Franke et al. [8], Denkena et al. [9], and Kaufmann et al. [10]. These systems rely on brushes, rollers or other *draping elements* that force the fabric to conform to the mold as the layup head advances. Additionally, in [10], an *adaptive material buffer* is introduced to compensate for the length differences of the two fabric edges created by the shear deformation.

The draping concepts by Schlangen [11] and Zhu et al. [12] enable to *pre-shear* the fabric in the layup head before it reaches the mold. This pre-shear can be used to control the distribution of shear angles in the fabric course and ultimately utilize the entire available shear reserve. The setup in [12] achieves the shearing through discrete *shifts*, i.e. by sequentially constraining and rotating the fabric in-plane to approximate curvature. The method in [11], on the other hand, continuously shears the fabric by means of drive wheels with varying rotational speeds. The latter will potentially be faster and result in less distorted fiber paths. However, only a small-scale stationary shearing device was tested in the study.

The draping behavior of a fabric is typically modeled using either a kinematic or mechanical approach. The extensional stiffness of the fibers is in the kinematic approach assumed infinite and the in-plane shear stiffness of the fabric is zero. The former assumption is justified by the high modulus of structural fibers, whereas the latter results from shear being the governing deformation mechanism during draping and that the shear stiffness, in general, is weak. With these assumptions, the fabric can be modeled as a grid of pin-jointed cells, see for instance Mack & Taylor [13] and Van der Weeën [14]. For moderate shear angles the draped configuration on the mold can be predicted with reasonable accuracy, and a low computational effort compared to the mechanical approach.

The mechanical approach is typically accomplished within the framework of the finite element (FE) method, see e.g. Liang & Boisse [15] and Fetfatsidis & Sherwood [16]. These models are generally recognized as giving more accurate draping predictions because they take the boundary and process conditions into account, and are typically based on a set of experimentally measured material data. On the other hand, they are computationally expensive to evaluate.

To control the movement of the layup head, some form of path planning algorithm must be devised. One approach is to employ a kinematic draping algorithm for calculating a feasible target configuration on the mold, including the necessary shear deformation [5], [9]. To this end, a framework was developed by the authors of the present study in which a kinematic draping model in combination with optimization is used to specify the individual courses in a laminate based on a structural layup plan [17]. The optimized courses have a tailored placement and shear distribution, in order to match criteria such as producibility, minimal material waste, and structural performance. The necessary information for generating the movement of the layup head can subsequently be extracted for each course.

The present study takes point of departure in the following research question: To what degree can the kinematically optimal paths generated with the framework [17] be realized with a layup head that can apply pre-shear in the fabric? To answer this question, a new layup head concept is presented which is based on principles from the reviewed literature. The system in focus enables continuous draping with a high deposition rate for molds of low complexity and thereby low shear angles. Further, the layup head must be able to drape courses with a varying shear distribution that follow a curved 3D path on the mold. The applicability of the concept is analyzed using a finite element (FE) model calibrated with experimentally determined material data for in-plane shear and out-of-plane bending. Using a simple parameter study, and draping sequences on different mold geometries, the concept is qualified for the subsequent physical prototyping.

The rest of the paper is organized as follows: Section 2 provides more details about draping in preforms and introduces the concept for an automatic pre-shearing draping tool. Section 3 presents the finite element model used for the virtual prototyping including the material model calibrated with experimental data. Section 4 presents numerical draping results and Section 5 and 6 round off the paper with a discussion and a conclusion.

## 2 Automatic draping in preforms

As a starting point, the background to automatic draping in preforms is outlined. This outlining involves a description of the fabric material and mold type, an elaboration of the benefits of pre-shearing and the method for generating the automatic draping sequences, and lastly, the criteria for a successful draping. Subsequently, the developed concept for automatic draping is presented along with design considerations.

### 2.1 Background and requirements

The fabric material utilized in this study consists of unidirectional (UD) glass fiber mats with its primary directions being in the 0^∘^-direction, which are stabilized with a layer of ±80^∘^ glass backing fibers. This configuration is sometimes denoted *quasi-UD*. The combined areal density of the UD layer and the backing layer is 1380 g/m^2^. The fibers are held together with a tricot-chain type stitching made from polyester. The total fabric thickness is approximately 1 mm. Through experimental characterization [18], it was found that the NCF material behaves equally in positive and negative shear, i.e. the direction in which a roving rotates. This means, for instance, that a shear angle of 4^∘^ will require the same magnitude of shear force to reach as a shear angle of -4^∘^.

For large composite structures such as wind turbine blades, the size of the mold necessitates multiple pieces of fabric material to achieve mold coverage. Typically, the fabric material comes in rolls of predefined widths, which thus form the basis for the manual layup process, as well as the layup systems for wind turbine blades reviewed in the introduction of this paper. A roll-width of fabric placed in the mold along a certain path is denoted a *course*. The layup process must be *continuous*, such that it can easily accommodate short as well as long courses. Furthermore, the layup head must be able to handle different roll widths.

**Figure 1:**
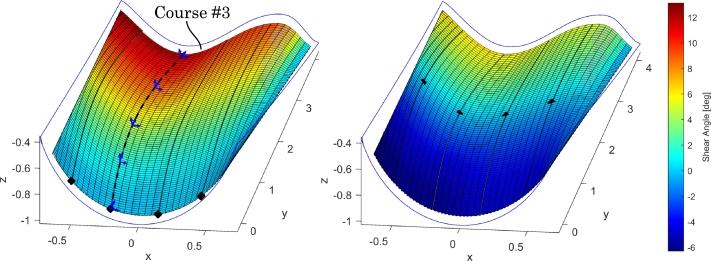
The effect of pre-shearing in a layer with five courses. Left: the courses start with 0^∘^ shear at the root end and the shear builds up to a maximum of 13^∘^ in the leftmost course. Right: the zero-shear point is moved towards the middle of the courses resulting in an approximately ±6.5^∘^ shear distribution. Course #3 is indicated, which will be used as a worst case test in Section 4. The shown shear angles are large compared to a typical wind turbine blade.

As mentioned in the introduction, pre-shear, i.e. that the course can be sheared before it reaches the mold surface, enables the utilization of the entire shear reserve of the fabric material. This concept is utilized in the course optimization framework [17] which uses a kinematic draping model for analysis [19], [20]. Fig. 1 presents an example of output from the framework, which highlights the opportunities of pre-shear: Recall, that the fabric material behaves equally in positive and negative shear. Therefore, by moving the point of zero shear, the same shear range of approximately 13^∘^ can be achieved, but only requiring a magnitude of 6.5^∘^ from the fabric material. This difference can determine whether a course can be draped or not, if the shear limit is exceeded.

For each course, the layup head tool path is provided by the course optimization framework. At the points defining the path, the local mold slopes and the amount of pre-shear is also provided. Notice though, that different strategies can be developed for determining the orientation of the layup head's tool center point (TCP) during draping. In this study, a simple strategy is chosen and implemented: the layup head is programmed to move tangentially to the left edge of the course being draped and with a rotation about the longitudinal *y*-axis, that follows the average mold slope in the width-direction of the course. The left edge of course #3 is indicated in Fig. 1 as a black dashed line along with some local coordinate systems of the TCP. A suitable offset normal to the mold must also be specified to avoid collisions between the layup head and the mold.

**Figure 2:**
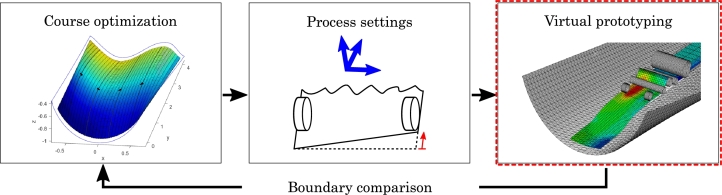
Workflow of the study: Individual courses are specified through an optimization framework with a kinematic draping model (a previous study [17]). Results from the framework provide the background for computing the process settings for the layup head, i.e. location and orientation of layup head as well as pre-shear. The ability of the layup head to realize the optimal courses is tested through virtual prototyping with a FE model (main focus of this study). Subsequently, the FE results are compared with the kinematic courses.

The output from the kinematic model functions as the target when draping a course and it can, therefore, also be re-employed when assessing the concept of the layup head. That is, whether the layup head can drape a course that follows the boundaries of the kinematic prediction. The kinematic model is, however, not without limitations and basically only predicts a final configuration on the mold without taking the draping process into account. A further criterion for a successful draping, is therefore, that the courses can be draped free from wrinkles, which could otherwise weaken the mechanical properties of the composite part. The workflow of the paper is presented graphically in Fig. 2.

### 2.2 Concept for automatic draping

**Figure 3:**
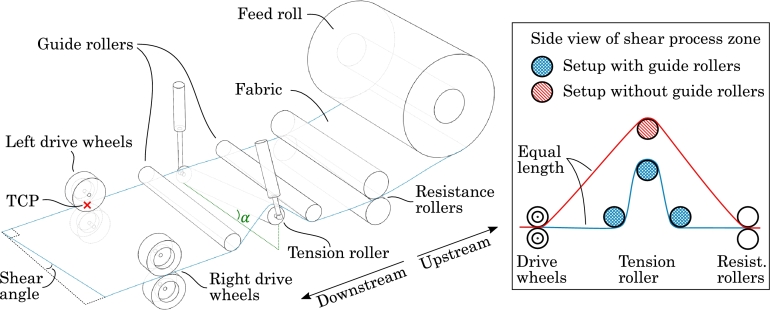
Concept for layup head with the ability to pre-shear the fabric. In the shown configuration, the right drive wheels have moved slower than the left drive wheels, thus inducing shear. The excess material is taken up by the tiltable tension roller, which is tilted by the angle *α*. Notice the local directions, i.e. left, right, upstream and downstream.

Based on the literature review and the requirements of continuous draping as well as the ability to pre-shear the fabric material, a layup head concept was developed which is presented in Fig. 3. The overall idea is to let *drive wheels* advance the fabric material forward and by adjusting their relative rotational speeds, shear can be induced, i.e. by letting one edge of the fabric travel faster than the other. This mode of operation is analogous to the concept by Schlangen [11]. To take up slack in the material when shear builds up, an adaptive material buffer analogous to that presented by Kaufmann et al. [10] is added, here denoted the *tension roller*. As it will become evident with the presented results in Section 4, not only does the tension roller take up slack but it plays an active part in the generation of shear. The individual components of the concept will be described in the following.

Starting the description upstream, the glass fiber fabric is unwound from the feed roll, i.e. the material roll from the supplier, which thus does not require prior handling. The fabric then continues between the resistance rollers, which clamp the fabric and applies resistance through a braking torque on the rollers. Another purpose of the resistance rollers is to prevent induced shear from reaching the feed roll, effectively creating a shear process zone between the resistance rollers and the drive wheels.

Next, the fabric is led under the first *guide roller*, over the tiltable tension roller and under the second guide roller. Either side of the tension roller can be lifted with a force controlled actuator, thus creating the tilt that tensions the fabric. When the tension roller tilts, there is a risk of the fabric sliding down the slope of the roller and for this reason, the necessary tilt angle, *α*, should be kept as low as possible. The guide rollers serve to reduce this tilt angle as seen in the right side of Fig. 3, but it was furthermore found that the slight added friction from these rollers also helps to keep the fabric on track.

The last group of components on the layup head concept are the four drive wheels, i.e. with a pair of wheels placed on either side of the fabric. Analogous to the resistance rollers, the drive wheels clamp the fabric to achieve enough traction to induce the shear. The drive wheels are velocity controlled, such that the amount of shear can be regulated. The feed of fabric material from the layup head must, however, also be synchronized with the movement of the layup head relative to the mold.

In terms of handling different roll widths, the respective rollers can be made sufficiently wide to handle the maximum width. The drive wheels, however, must be adjustable in the width direction such that they are located on the edge of the fabric. As can be seen from Fig. 3, the layup concept can, theoretically, create a linearly varying difference in position across the width of the fabric, which means a constant shear angle at each cross section. By observing the kinematically draped courses in Fig. 1, it can be concluded that the shear angles are mostly constant at each cross section. The investigations in this study can clarify if the shear complexity of the layup head concept suffices. Notice, that the presented concept does not have a draping element, i.e. rollers or brushes, on the mold. This topic is revisited in Section 5.

## 3 Draping process model

The numerical simulation model used for assessing the layup head concept is developed in the commercial FE code Abaqus. It is a transient nonlinear multi-body 3D model solved using explicit time integration due to many nonlinearities. The model has rigid body representations of the different parts of the layup head and using so-called *connector elements*, their kinematic behavior can be controlled [21]. The connector elements enable control of local degrees of freedom (DOF) of the reference point of a rigid body. The fabric material is modeled using the ABAQUS *fabric* material model [22]. This material model is developed for fabrics with two fiber directions and stress-strain data is required as input. The deformation modes include fiber tension and compression as well as in-plane shear.

First, the geometry and kinematics of the layup head model is outlined and afterwards the material model is described including the experimental tests conducted for calibration. Lastly, the assignment of boundary conditions and contact definitions are explained.

### 3.1 Geometry and kinematics

**Figure 4:**
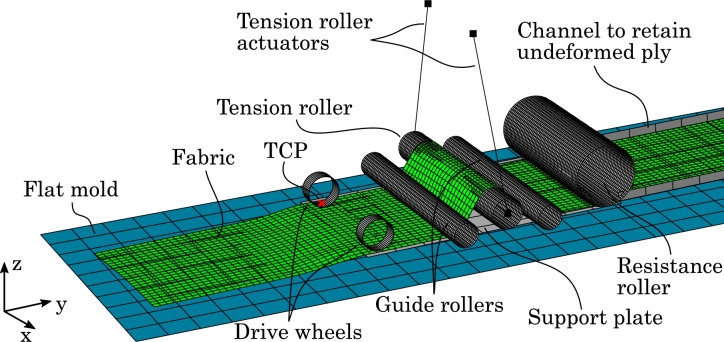
Finite element model of the layup head (gray) draping a fabric (green) on a flat mold (blue).

Following the concept sketch in Fig. 3, a representative FE model is created with some simplifications, which will be discussed in the following. The finite element model can be seen in Fig. 4. The main layup head components are the resistance roller, the tension roller and guide rollers, and the drive wheels, all of which are attached to a support plate that constitutes the structure of the layup head.

**Figure 5:**
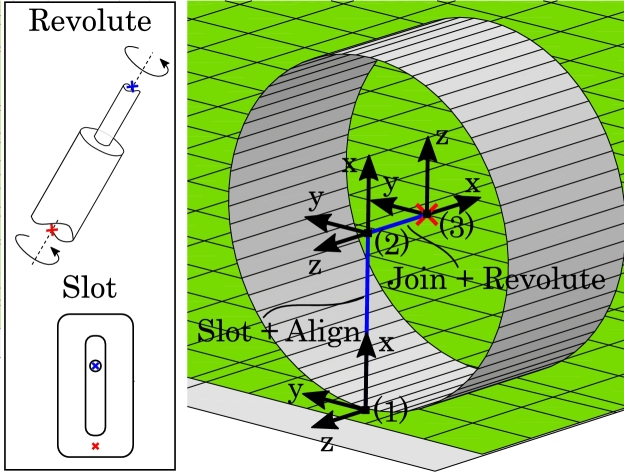
Connector elements to control the kinematics of a drive wheel. The red cross indicates the reference point of the drive wheel which is located in its geometrical center. The Join+Revolute connector allows for spin around the axis between point (2) and (3). The Slot+Align connector allows for translation along the axis between point (1) and (2). Point (1) is fixed to the support plate.

Starting downstream, the drive wheels must be able to shear the fabric and thus require a high traction with the fabric. For this reason, in the concept sketch (Fig. 3), the drive wheels are mounted in pairs such that the fabric can be clamped. In the FE model, the left and right upper drive wheel will instead press down on the frictionless support plate. Each drive wheel is modeled as a rigid outer shell and makes use of two types of connector elements as seen in Fig. 5. The first is a *Join+Revolute*-type connector which fixes the translational DOF and two rotational DOF but allows for rotation about the axis of the drive wheel. This connector will enable the assignment of the rotational velocity boundary conditions to the drive wheel. The second connector element is a *Slot+Align*-type which fixes all rotational DOF and two translational DOF but allows for translation along a local axis normal to the support plate. This connector will enable the assignment of a force that clamps the fabric between the drive wheel and the support plate, which along with the contact definition, will create the necessary traction.

The tension roller and the two guide rollers must be able to tension the fabric by taking up slack stemming from shearing. Recall that shear is generated by letting one side of the fabric travel faster than the other. The fabric material just needs to slide over these rollers so in principle they could be modeled as fixed in axial rotation and frictionless. It was, however, found that including friction and the ability of spinning was quite important to keep the fabric from sliding down the slope of the tension roller when it tilts. Therefore, a Join+Revolute connector is installed for each of the two guide rollers analogous to that in Fig. 5. The second point of the connector elements and thereby the translations of the guide rollers are fixed relative to the support plate. The tension roller is likewise allowed to spin via a Join+Revolute connector and in addition, each of its two ends are attached to a connector element that resembles the linear actuator (see Fig. 4). These connectors have an added stop such that they cannot extend beyond the initial length (full stroke) as well as velocity proportional damping. Because the fabric shears, there will be relative movement between the fabric and the tension roller and guide rollers. For this reason the friction on the rollers can not be too high as this would inhibit the shearing.

The resistance roller ensures an even tension in the fabric material downstream, and prevents any induced shear from moving to the feed roll. The resistance roller is modeled similarly to a drive wheel, i.e. with the outer shell of the upper roller and a Join+Revolute connector to enable spin and a Slot+Align connector to enable movement normal to the support plate. These two DOF allow the assignment of respectively the braking torque and the clamping force.

**Figure 6:**
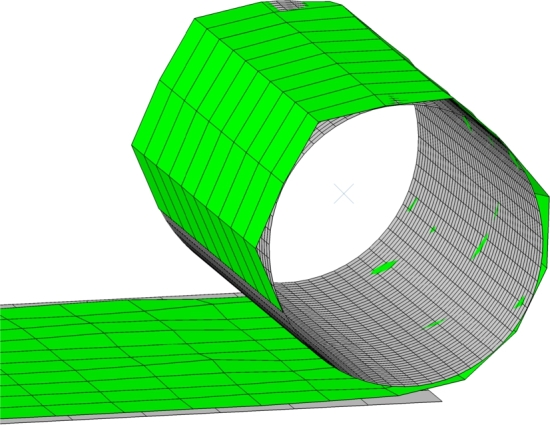
Winding fabric material with a coarse mesh onto the feed roll. This modeling technique was discarded.

The fabric material being draped will be fed from a stock roll of glass fiber fabric. This part of the layup head is not straightforward to model and requires some extra attention. Basically, the deformable parts in an FE model are typically created in a stress-free, undeformed configuration, which thus differs from a fabric rolled multiple times over itself on the feed roll. A number of solutions have been conceptualized: The first solution involves winding of the fabric onto the feed roll in a separate analysis step. In Abaqus, it is possible to continue an analysis from a previously saved output database and the winding step would thus only have to be computed once. Winding the fabric onto the feed roll without crumpling the mesh, however, requires a very fine discretization and a low winding speed which would thus result in very long solution times. An example of winding with a too coarse mesh can be seen in Fig. 6. The feed roll geometry could also be created in the deformed configuration with added pre-stress, but the mesh discretization must still be low. Another approach is to discard the feed roll and have all the fabric initialized in an undeformed, flat configuration. Using *progressive element activation* (element birth), only the relevant fabric elements could be made active during the analysis. This feature is, unfortunately, not available in Abaqus Explicit. A simpler approach with the entire fabric length initialized is to keep the elements active and retained behind the resistance roller in an extended part of the support plate. The downside of the latter approach is the potential collisions with the mold as well as keeping the fabric undeformed during movement of the layup head, but the approach is chosen for the assessment of the concept in this study.

**Figure 7:**
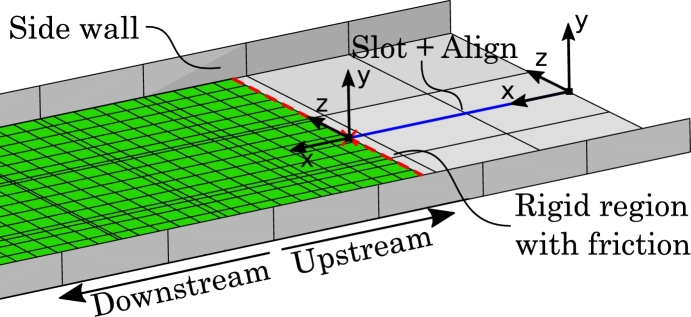
Extended support plate (upstream of resistance roller) with channel to retain undeformed fabric. The red dashed line indicates the rigid fabric end with added friction.

To retain the fabric upstream of the resistance roller, a number of features have been implemented in the model as seen in Fig. 7: Side walls have been added to the extended support plate, effectively creating a channel that guides the undeformed fabric. Also, the end of the fabric has been made rigid and a Slot+Align connector has been added such that the fabric end only can move forward within the channel. Friction between the fabric end and support plate has been added to further retain the fabric. Notice, that these features only are modeling artifacts.

Regarding the size of the layup head model, the shear process zone, i.e. distance between the resistance roller and the drive wheels is 750 mm. Details of the dimensions can be found in Appendix A with some changes being tested throughout the results in Section 4. The widths of the fabrics considered are 350 mm and 500 mm, with the width of the support plate and the position of the drive wheels being changed accordingly. The fabric thickness is 1 mm.

### 3.2 Material model

As mentioned in the beginning of the section, the simulation model uses the Abaqus *fabric* material model. The input to the material model comes from experimental coupon tests. The fabric material considered in this study has previously been characterized in terms of shear [18] and out-of-plane bending [23]. The shear response was characterized using the bias-extension test, i.e. with a test specimen being elongated in a universal testing machine with the fibers initially oriented at 45^∘^ to the loading direction. The bending stiffness was characterized using a variety of different methods, among others the Peirce's cantilever test, i.e. by measuring the maximum deflection of a strip of fabric deflecting under the action of gravity. Please see the references for more details.

Along with the shear characterization in [18], a membrane FE simulation model was developed to successfully verify that the experimental crosshead force vs. displacement of the bias-extension sample could be captured with the material model. This membrane material model is enhanced in the present study to also account for the out-of-plane bending with the already characterized bending stiffness. The bending stiffness is necessary for an accurate representation of potential wrinkles [24]. Additionally, permanent deformation characteristics are measured through bias-extension tests with unloading and reloading, and are also implemented in the material model. The motivation for this model enhancement is that the fabric will experience loading and unloading when being handled by the layuphead.

**Figure 8:**
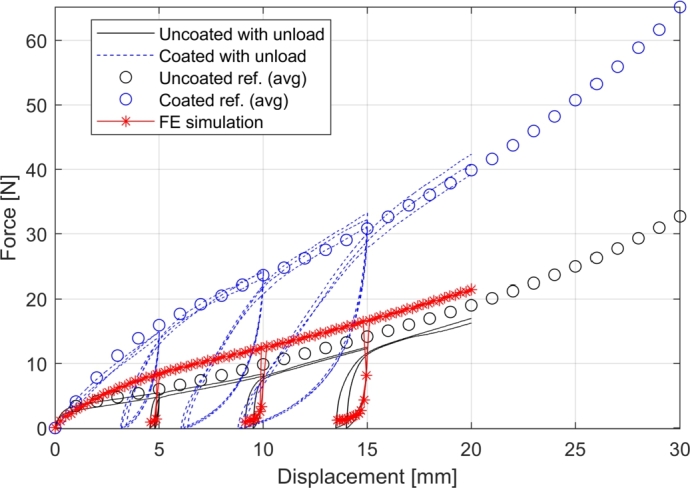
Crosshead force vs. displacement curves for bias-extension test with unloading and reloading. The circles are averaged reference data with monotonic loading.

In the previous experimental characterization studies on shear and bending [18], [23], the fabric was coated with a binder material that enables pre-consolidation of the stack. In the same manner as described in those references, the fabric material without the binder was also characterized. It was found that the shear characteristic was significantly affected by the binder and that, on average, the stiffness was approximately twice as high compared to that without the binder. The results from the bias-extension tests with unloading and reloading in Fig. 8 also suggest that the binder-coated fabric has significantly more springback, i.e. that there is less permanent deformation. The figure presents the crosshead data from the testing machine. The setup was a sequence of loading, unloading to 0 N and reloading at 10 mm/min. The unloading took place when the crosshead reached 5, 10 and 15 mm of displacement, respectively, which corresponds to shear angles of approximately 2^∘^, 5^∘^ and 8^∘^. Notice, that the monotonic force-displacement data are processed to shear stress vs. shear angle data for inclusion in the material model. Further, based on the shear angles measured in the unloaded states, an average *unload modulus*, $U_{\mathrm{shear}}$ can be computed. Shear unloading and re-loading in the FE model will occur with that modulus.

**Figure 9:**
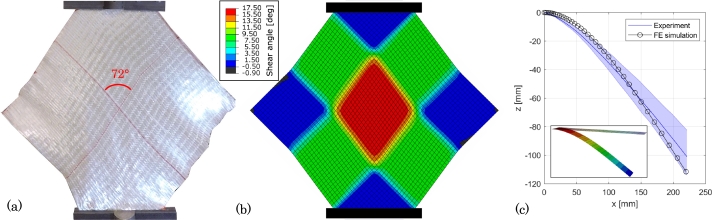
Verification of the FE material model. (a) Experimental diamond-shaped bias-extension specimen at 20 mm elongation (initial gripper distance: 270 mm, clamped width: 120 mm). The average shear angle in the center region is 90^∘^−72^∘^=18^∘^. (b) FE simulation of bias-extension test at 20 mm elongation. (c) Comparison of experimental and FE deflection of cantilever bending test with 50 mm × 250 mm specimen. The shaded area indicates the range of the experimental data. The deformed FE model is seen inserted in the graph.

For the reasons above it was decided to develop the layup head concept for draping the uncoated fabric material and apply the binder in a subsequent process step. As a verification of the material model, consider the results from a bias-extension test simulation with unloading and reloading presented in Fig. 9 (a) and (b), with the simulated crosshead reaction force plotted in Fig. 8. The FE model is seen to capture the in-plane shear part of the UD fabric behavior reasonably well. Notice, that the FE model initially was too stiff in shear because of artificial strain energy. It was found that the default computed value of the penalty stiffness of the drilling DOF of the shell element was too high. A decrease factor of 100 was found adequate.

The out-of-plane bending stiffness is added to the model by first substituting the membrane elements for shell elements (4-noded with reduced integration and hourglass control). With these standard structural elements, however, the bending response is calculated from the membrane properties through shell theory, which is not applicable to fabric materials. The remedy employed in this study is a decoupling achieved by means of three fictitious layers in the shell as proposed by Döbrich et al. [25]. The thin inner layer (1/8 of the total thickness) will mostly account for the membrane stiffness and the two outer layers will mostly account for the out-of-plane bending stiffness. Each layer has a constant stiffness in the 1 (UD roving) and 2 (backing) material directions. With a target membrane and bending stiffness for the combined shell, the stiffnesses for each layer can be computed using classical lamination theory. The target membrane stiffnesses were chosen as a balance between obtaining a sufficiently low strain in the fiber directions and getting a stable time increment. Recall that the stable time increment in explicit time integration scales inversely with the stiffness of the material therefore, simplification can be made to reduce the computational time [26]. Membrane stiffnesses of, respectively, 5.0 $\times 10^{9}$ Pa (1-direction) and 6.0 $\times 10^{8}$ Pa (2-direction) were found adequate. The target bending stiffnesses used in the model are processed from experiments [23] and are, respectively, 5.0 $\times 10^{-2}$ Nm^2^ (1-direction) and 8.7 $\times 10^{-4}$ Nm^2^ (2-direction). Notice that the latter value had to be increased by a factor of 10 to achieve numerical stability but in spite of this, it is still two orders of magnitude lower than the bending stiffness in the 1-direction. The comparison of data from cantilever bending test and FE simulation is presented in Fig. 9(c). The deflection is seen to be captured reasonably well, even though the bending stiffness is approximated as constant, i.e. independent of curvature, in the model. The input data to the material model are provided in Table 1. Further, global mass-proportional Rayleigh damping is employed.

**Table 1:** Data input to fabric material model. The shear stress as function of shear angle, *τ*(*γ*) can be recreated with the polynomial fit and is the same for all three layers.

	Middle layer	Top/bot. layer
*E*_1_	3.63 ×10^10^ Pa	5.30×10^8^ Pa
*E*_2_	4.79 ×10^9^ Pa	1.10×10^6^ Pa
*τ*(*γ*)	$\sum_{i=1}^{8}p_{i}\gamma^{i}$ Pa, p= [0.00725, -0.0878, 0.622, -2.17, 3.48, -1.28, -2.48, 2.03$]\times 10^{8}$, 0≤γ≤0.61 rad	
*U*_shear_	1.0 $\times 10^{8}$ Pa	

### 3.3 Boundary conditions and contact

A variety of boundary conditions are assigned to the layup head model. First of all, the mold remains fixed during the analysis. As discussed in Subsection 2.1, the layup head will be controlled based on output from a kinematic draping analysis, see e.g. Fig. 1. The reference point of the support plate and thereby the TCP (tool center point) of the layup head, is located under the left drive wheel, which enables to steer the layup head after the left course edge, and follow the slope of the mold by rotation about the *y*-axis. The tangential speed of the left drive wheel is synchronized with the speed of the layup head and the right drive wheel will be either slower or faster depending on the amount of shear that needs to be induced. At any given point along the length of the course, the position difference between the left and right fabric edge, which is created by the difference in the wheel speeds (see equation (1)).

(1) $$\Delta_{i}=\sin(\overset{¯}{\gamma_{i}})\;W_{\mathrm{fabric}}$$

Here $\overset{¯}{\gamma_{i}}$ is the average shear across the width of the fabric at the given point *i* and $W_{fabric}$ is the width of the fabric. The time-varying boundary conditions are assigned using *amplitudes* in Abaqus.

The tension roller and resistance roller are, respectively, force and torque controlled. The balance must be such that the stretching force exerted on the fabric by the tension roller must be less than that exerted by the braking torque of the resistance roller. If this is not the case, the tension roller and not the drive wheels will “unwind” the fabric. On the other hand, the force from the tension roller must be large enough to properly tension the fabric and to even out local shear deformation created by the drive wheels. Through numerical experimentation, the tension roller lifting force was set to 100 N and the braking torque of the resistance roller to 8 Nm. Additionally, a velocity-proportional damping was added to the tension roller lift connector to remove oscillations. During this numerical experimentation, it was also found that the tension roller had to be raised up evenly in both sides relative to the guide rollers before the draping step (compare the tension rollers and guide rollers in Figure 3, Figure 4). As it turns out, the guide rollers and tension roller effectively work like a pulley system and only with this initial raising does the gearing ratio approach the expected value of 0.5. A non-constant gearing ratio would mean that the stretching force exerted on the fabric by the tension roller would be dependent on the tilt angle which would inhibit passive force control.

General contact with possibility for re-separation (as opposed to sticky friction) is assigned to all surfaces with which the fabric is in contact. Coulomb friction is added to all contact pairs except the fabric and support plate-pair. The coefficient of friction with, respectively, the drive wheels and the resistance roller, is set to the unrealistically high value 100 in order to prevent any slippage (along with a 100 N clamping force). The coefficient of frictions with the guide rollers and the tension roller are both 0.2 and for the mold it is set to 0.1.

## 4 Numerical draping results

The applicability of the layup head for draping quasi-UD glass fiber fabric is assessed in this section using the FE model presented in the previous section. Four different test cases are considered:

1. The effect of the tension roller.
2. C-shaped course on flat mold.
3. Constant-shear straight course on flat mold.
4. Course on double-curved mold.

Case 1. is without a mold to illustrate the effect of the tension roller using a split roller design. Case 2. is a C-shaped course on a flat mold which, due to its shape, results in kinematic shear angles that are constant across the width and ranges from 0^∘^ at the start and 5^∘^ at the end. This course will test the layup head's ability to drape along a steering curve and induce varying shear along the length of the course. Because the mold is flat, pressing the fabric onto the mold does not induce shear and the shear development is thus completely decoupled from the mold curvature. Case 3. is likewise on a flat mold but involves a straight course with 5^∘^ constant shear in the entire course. It will test the ability to pre-shear and maintain this pre-shear throughout the length of the course. Again, the shear development is decoupled from the mold and further, also decoupled from the path of the layup head. Case 4. makes use of the industrially relevant mold in Fig. 1 and the indicated course #3. As it can be seen from the figure, the course starts with 0^∘^ shear which builds up to approximately 10^∘^ at the end. The course curves in 3D and the shear is non-constant across the width of the course, which will challenge the layup head. All three courses draped on a mold, i.e. case 2.-4. are 4 m long. The element size is approximately 20 mm.

### 4.1 The effect of the tension roller

**Figure 10:**
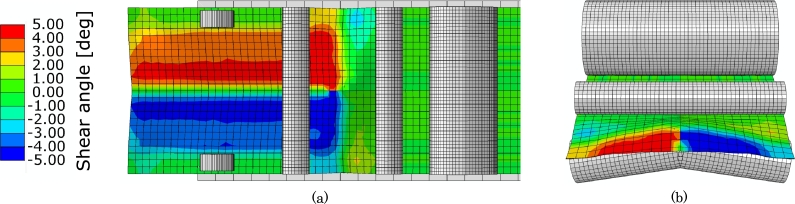
Numerical result with a double tension roller. (a) Top view of FE model with shear angle color contours. (b) Front cut view.

The results from the first test case without a mold are presented in Fig. 10a and -b. The purpose of this test case is to show the influence of the tension roller on the shear deformation of the fabric, and also explore a different design configuration. To this end, the single tension roller design shown with the FE model in Fig. 4 has been upgraded to a double split roller design. This design change enables to kink the tension roller at the middle, thus creating the deformation seen in the cut view in Fig. 10(b). The drive wheels in this analysis are advancing the fabric with an equal velocity, thus not inducing any shear. Therefore, only the tension roller is responsible for creating the approximately ±5^∘^ shear deformation seen in Fig. 10. It can therefore be concluded that the tension roller, whether single or double, plays an active part in the shear creation. Notice, that the remaining test cases will make use of a single tension roller design but the topic is discussed further in Section 5.

### 4.2 C-shaped course on flat mold

**Figure 11:**
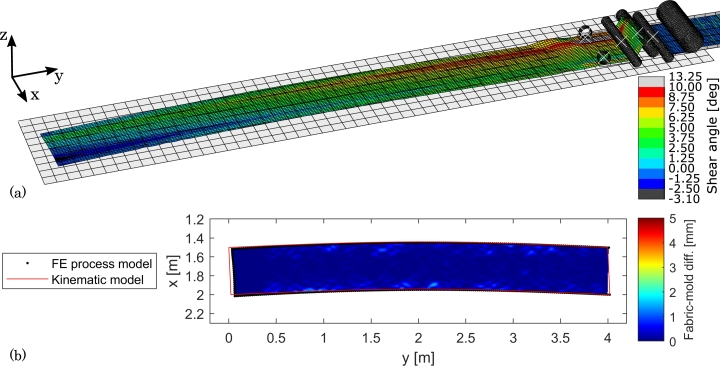
Numerical results from draping a 4 m C-shaped course on a flat mold. (a) 3D view of FE model with shear angle color contours. (b) Top view of fabric-mold difference and comparison to fabric boundary from kinematic model.

The results from the C-shaped course on the flat mold are presented in Fig. 11. From part (a) of the figure it can be seen that the shear distribution across the width of the course not is constant as the kinematic model predicts. Rather, it appears that the drive wheels result in local deformations. For instance, at the course start, which should have zero shear, the shear angles next to the left drive wheel reach 3^∘^ whereas the shear angles next to the right drive wheel reach -3^∘^. The average shear angle across the width of the course does, however, match the kinematic prediction well in that it starts at approximately 0^∘^ and increases monotonically to approximately 6^∘^ after 4 m draped length. It can be observed that a hump of excess material has formed just downstream of the left drive wheel, which indicates that too much material is fed in this side. The issue appears to be the tension roller width and the hinging at its ends: The setup is such that the width of the tension roller (720 mm) is larger than the width of the fabric (500 mm), i.e. to accommodate wider fabrics, but the fabric is centered on the tension roller. Therefore, the point on the tension roller where the left fabric edge is in contact, will also move up when the tension roller tilts which will thus violate the kinematics.

From part (b) of Fig. 11, it can be seen that the fabric conforms closely to the mold, in spite of the shear discrepancy with the kinematic model. There appears to be some insignificant local wrinkles close to the fabric edges which are probably due to the drive wheels and their interaction with the fabric. The boundary from the kinematic model is traced well overall, but the start of the course has a discrepancy of up to 48 mm. From the intermediate results of the FE analysis, the fabric is observed to slide on the mold as the layup head advances, which is assessed to be the cause of the boundary discrepancy.

### 4.3 Constant-shear straight course on flat mold

**Figure 12:**
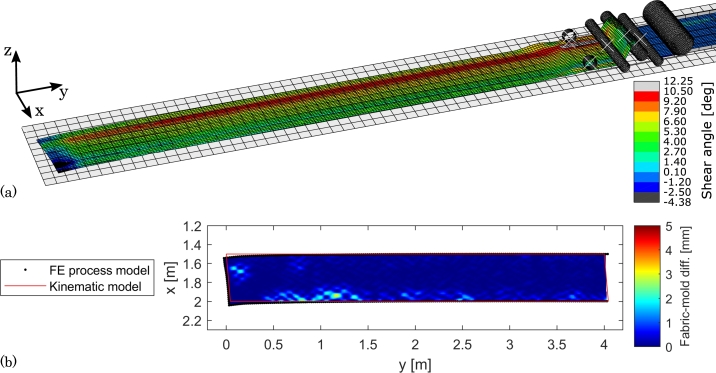
Numerical results from draping a 4 m straight course with constant shear on a flat mold. (a) 3D view of FE model with shear angle color contours. (b) Top view of fabric-mold difference and comparison to fabric boundary from kinematic model.

The results from the straight constant-shear course on the flat mold are presented in Fig. 12. Through the development of the model, it was found challenging to create instant pre-shear of the fabric without creating wrinkles. It was therefore decided to build up the pre-shear over a distance of 100 mm, before continuing the layup head with this constant shear. From Fig. 12(a) it can be seen how the shear builds up, and afterwards is maintained more or less constant along the course length. The distribution across the width is, as in the previous case (Subsection 4.2), still non-constant, i.e. with local deformation from the drive wheels, and the same hump of excess material is visible, which suggests the same issue with the tension roller width.

Fig. 12(b) shows that the mold conformation is good, but with some minor wrinkles observed close to the fabric edges as previously. Slightly larger wrinkles are visible at the first part of the course, which could be due to the challenge with building up of the pre-shear. Notice though, that the shape of the predicted wrinkles will be somewhat mesh dependent. In the “steady-state” region, i.e. after the pre-shear build-up, the FE boundary matches the kinematic boundary well and the course remains straight. In the pre-shear build-up region there are boundary discrepancies up to 54 mm, which again are due to the fabric moving on the mold when the layup head advances.

### 4.4 Course on double-curved mold

**Figure 13:**
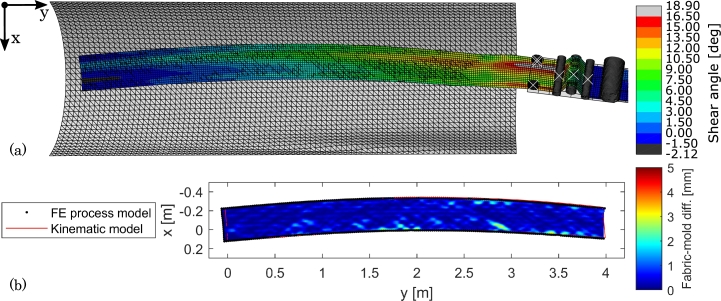
Numerical results from draping a 4 m curved course on a double-curved mold. (a) 3D view of FE model with shear angle color contours. (b) Top view of fabric-mold difference and comparison to fabric boundary from kinematic model.

The results with draping a course on the double-curved mold are presented in Fig. 13. Two issues, that were identified with the benchmark cases on the flat mold, are addressed in this analysis: the width of the tension roller, which generates an incorrect material feed and also the sliding on the mold. The width of the fabric in this analysis is 350 mm and the tension roller was therefore adjusted to a width of 400 mm such that there is only 50 mm excess width (in the previous cases the excess roller width was 220 mm). The fabric material is still centered on the tension roller. Regarding the sliding issue, the coefficient of friction between the fabric and the mold was increased from 0.1 to 1, i.e. a high value.

In Fig. 13(a) it is seen that there is still local deformations from the drive wheels but that the shear angles are more evenly distributed across the width of the course compared to test cases 2. and 3. The shear angles in Fig. 13(a) thus match those of course #3 in Fig. 1 reasonably well: The FE shear angles start at 0^∘^ and reach a average of approximately 10^∘^ at the course end. The fabric material now also leaves the layup head more evenly, i.e. without a hump. These observations suggest that the narrower tension roller in this test case is an improvement. Therefore, care must be shown in terms of the tension roller design such that it can accommodate different roll widths but achieving the desired slack uptake.

In Fig. 13(b) it can be seen that the mold conformity also is good with the usual small wrinkles at the course edges likely resulting from drive wheel interaction. The boundary is also traced well. The largest discrepancy (37 mm) is again at the beginning of the course but this time the course starts further back than the start of the kinematic boundary. The issue is therefore not sliding. Basically, the layup head TCP was offset 100 mm in the *z*-direction to avoid mold collisions due to the curvature of the mold. As a compensation, 100 mm extra fabric was fed through the drive wheels before the layup head began advancing. With the bending stiffness of the fabric, this compensation is, however, not straightforward and could need some tuning.

## 5 Discussion

The presented results give rise to a discussion about the applicability and potential improvements for the physical prototype. Overall, the presented results show promise. The first test case (Subsection 4.1) with the split tension roller design not only demonstrated its effect, but also served to explore a different design of the component. The current layup head concept can theoretically induce a constant shear distribution across the width of a course. In reality, local deformation from the drive wheels result in an uneven shear distribution. This limitation was, however, not giving rise to mold conformation issues in the test case on the double-curved mold (subsection 4.4), which according to the kinematic analysis has a slightly non-constant shear distribution across the width. The functionality of a varying shear distribution could maybe be relevant for even more curved mold geometries or wider courses.

For the presented test cases, the tension roller was force controlled, thus passively moving up and down according to the shear induced by the drive wheels. At the cost of more parameters to control in the layup head, the tension roller could also be displacement controlled, e.g. based on a simple kinematic model of the layup head. Still, some kind of sensor system for feedback control would probably be necessary, as even the slightest discrepancy between the fabric movement caused by the drive wheels, a displacement controlled tension roller and the layup head movement could lead to wrinkling.

One such discrepancy could arise with relative movement of the fabric between a set of drive wheels, i.e. slippage. In the presented test cases the coefficient of friction was unrealistically high, thereby neglecting any slip. Friction tests will be necessary to determine the amount of available traction and ultimately the magnitude of shear angles that can be induced. In the bias-extension tests with the fabric material, shear angles of 30^∘^-40^∘^ could be reached, but the tested coupon size was relatively small compared to a general course and also the boundary conditions were different. Also, potential damage to the fabric from the drive wheel interaction could be investigated.

Another issue that was experienced with the test cases was sliding of the already draped fabric on the mold, as the layup head advanced. In the fourth test case with the double-curved mold (subsection 4.4), the issue was mitigated with an increased mold friction. To this end, it could be investigated if the entire mold needs to have higher friction, or if it is only an issue in the beginning of the courses. An increased mold friction is also highly relevant on the steeper parts of the mold, i.e. where a component of gravity tangential to the mold surface can cause sliding. Different concepts for controlling the mold friction, i.e. mechanical or chemical, can be tested, but care must be shown not to influence the draping or subsequent infusion process.

Lastly, the physical layup head prototype should be made more compact, and, preferably, vertically oriented, to alleviate potential issues with mold collisions. Another addition could be a *draping element*, i.e. rollers or brushes that smooths the fabric on the mold. Such a feature could maybe even make it up for a potential complex tension roller design on more curved mold geometries. More drive wheels across the width of the fabric could also be considered, e.g. as a remedy to increase the traction, even out local deformation from the drive wheels, and also potentially handle fabrics that have been pre-cut in the width. However, for all these additions, the benefits should be weighed against the added complexity and mass.

## 6 Conclusion

A concept for automatic draping on double-curved surfaces of wide fabrics has been presented and analyzed using a virtual prototype. The concept was synthesized from technological elements described in the literature, of which the drive wheels and tension roller (adaptive material buffer) are the principle components. The concept can produce a continuous layup of fiber fabric from a feed roll, while applying pre-shear to the fabric before it reaches the mold. This ensures that optimal fabric courses of arbitrary length can be handled.

The virtual prototype is formulated as an advanced finite element (FE) model with rigid multi-body kinematics, and a fabric material model calibrated with experimental coupon tests. The analyses performed with a variation of tension roller design, draping on a flat mold and on a double-curved mold, provided valuable insight into the working principle and performance of the concept. Drawbacks and potential improvements for the physical prototype were subsequently discussed, among others the present constant shear distribution across the width of a course, and whether it needs to be more complex.

Overall, it is concluded that the presented concept with a few modifications can become a valuable tool for automating the draping process for large composite structures, and thereby lower the associated costs. With a finished production-ready draping system, the FE model could be upgraded to an offline quality inspection tool.

## Funding

This study was carried out as part of the MADEBLADES research project supported by the 10.13039/501100022591Energy Technology Development and Demonstration Program, Grant no. 64019-0514.

## CRediT authorship contribution statement

**Christian Krogh:** Conceived and designed the experiments, Performed the experiments, Analyzed and interpreted the data, Contributed reagents, materials, analysis tools or data, Wrote the paper.

**Peter H. Broberg; Sebastian M. Hermansen; Asbjørn M. Olesen:** Conceived and designed the experiments, Analyzed and interpreted the data, Wrote the paper.

**Brian L.V. Bak; Esben Lindgaard; Erik Lund; Jørgen Kepler; Johnny Jakobsen:** Conceived and designed the experiments, Analyzed and interpreted the data.

## Declaration of Competing Interest

The authors declare the following financial interests/personal relationships which may be considered as potential competing interests: Johnny Jakobsen reports financial support was provided by 10.13039/501100022591The Energy Technology Development and Demonstration Programme, Grant no. 64019-0514. Christian Krogh reports financial support was provided by 10.13039/501100022591The Energy Technology Development and Demonstration Programme, Grant no. 64019-0514.

## Figures and Tables

**Table A.2:** Dimensions and locations of wheels and rollers in FE model of layup head. The *y*-coordinates are given relative to the TCP. The guide rollers are centered around the tension roller lengthwise with a spacing of 280 mm.

Component	y coor. [mm]	Radius [mm]	Width [mm]
Drive wheels	0.0	50	50
Guide roller, downstr.	235	40	720
Guide roller, upstr.	515		
Tension roller	375	50	720
Resistance roller	750	100	*W*_fabric_ + 40 mm

## Data Availability

Data will be made available on request.

## Appendix A Dimensions of layup head FE model

This appendix provides the baseline dimensions of the layup head model (changes are explicitly stated for each test case). The width of the support plate and also the width-wise location of the drive wheels is adjusted based on the fabric width being draped. The drive wheels are placed with an inset of 35 mm in *x* and 50 mm in *y*, measured from the wheel center to the fabric edges in each side. The different rollers are always centered width-wise with respect to the support plate. The *y*-coordinates, radii and widths of the wheels and rollers are presented in Table A.2.
